# Holocene geochemical footprint from Semi-arid alpine wetlands in southern Spain

**DOI:** 10.1038/sdata.2018.24

**Published:** 2018-02-27

**Authors:** Antonio García-Alix, Francisco J. Jiménez-Espejo, Gonzalo Jiménez-Moreno, Jaime L. Toney, María J. Ramos-Román, Jon Camuera, R. Scott Anderson, Antonio Delgado-Huertas, Francisca Martínez-Ruiz, Ignasi Queralt

**Affiliations:** 1 Departamento de Estratigrafía y Paleontología, Universidad de Granada, Granada 18071, Spain; 2 School of Geographical and Earth Sciences, University of Glasgow, Glasgow G12 8QQ, UK; 3 Instituto Andaluz de Ciencias de la Tierra (IACT), CISC-UGR, Armilla 18100, Spain; 4 Department of Biogeochemistry (JAMSTEC), Yokosuka 237-0061, Japan; 5 School of Earth Sciences and Environmental Sustainability, Northern Arizona University, Flagstaff, AZ 86011, USA; 6 Institute of Environmental Assessment and Water Research (IDAEA), CSIC, Barcelona 08034, Spain

**Keywords:** Limnology, Geochemistry, Environmental chemistry, Limnology, Palaeoclimate

## Abstract

Here we provide the geochemical dataset that our research group has collected after 10 years of investigation in the Sierra Nevada National Park in southern Spain. These data come from Holocene sedimentary records from four alpine sites (ranging from ∼2500 to ∼3000 masl): two peatlands and two shallow lakes. Different kinds of organic and inorganic analyses have been conducted. The organic matter in the bulk sediment was characterised using elemental measurements and isotope-ratio mass spectrometry (EA-IRMS). Leaf waxes in the sediment were investigated by means of chromatography with flame-ionization detection and mass spectrometry (GC-FID, GC-MS). Major, minor and trace elements of the sediments were analysed with atomic absorption (AAS), inductively coupled plasma mass spectrometry (ICP-MS), as well as X-ray scanning fluorescence. These data can be reused by environmental researchers and soil and land managers of the Sierra Nevada National Park and similar regions to identify the effect of natural climate change, overprinted by human impact, as well as to project new management policies in similar protected areas.

## Background & Summary

Arid Mediterranean ecosystems, especially alpine wetlands, are particularly vulnerable to climate oscillations, and their management and protection requires a complete knowledge of their response to past natural climate fluctuations and human-induced biochemical changes^1–4^. Recent works in the protected Sierra Nevada National Park in southern Spain have shown that the environmental evolution of neighbouring alpine wetlands can develop different sensitivities and long-term environmental responses during the Holocene, regardless of similar natural forcings^4–7^. This feature, which is common in areas under extreme climate conditions, supports the importance of datasets like those described here to understand past, present and potential future behaviours of vulnerable areas under similar climate and human pressure.

Additional climatic stresses exist for high altitude alpine wetlands in the Sierra Nevada, which are covered by snow from ∼November to ∼April. Due to limited access, only few and irregular meteorological records have been collected since 1960 (http://www.aemet.es/es/datos_abiertos/AEMET_OpenData, http://linaria.obsnev.es/). As an example, an observatory at 2500masl registered mean annual temperatures (MAT) of ∼4.4 °C and annual precipitation of ∼750 mm from discontinuous records between 1965 and 1993 (http://www.aemet.es/es/datos_abiertos/AEMET_OpenData, http://linaria.obsnev.es/). Meteorological data are even more scarce at higher elevations. Data collected from ~3020masl recorded a MAT of ~2.8 °C in the 2000 s (http://linaria.obsnev.es/) while additional collections at ~3100masl registered a MAT of 2.1 °C in 2016 (http://www.mapama.gob.es/es/red-parques-nacionales/red-seguimiento/). Although precipitation and isotopic records are rare at these elevations, monitoring programs from 2001 to 2003 succeeded in measuring isotopes from precipitation (snow) between 1030 and 3020masl (δD=−111.9±12.7‰ and δ^18^O=−16.1±1.9‰)^8^. These values are much lower than the ones at lower elevations in the south of the Iberian Peninsula^9^. Similarly, scarce data from the alpine lakes show mean δ^18^O values of −7.7±1.8‰, although they can reach −4.5‰ due to evaporative process in the shallowest lakes^10^.

**Figure 1:**
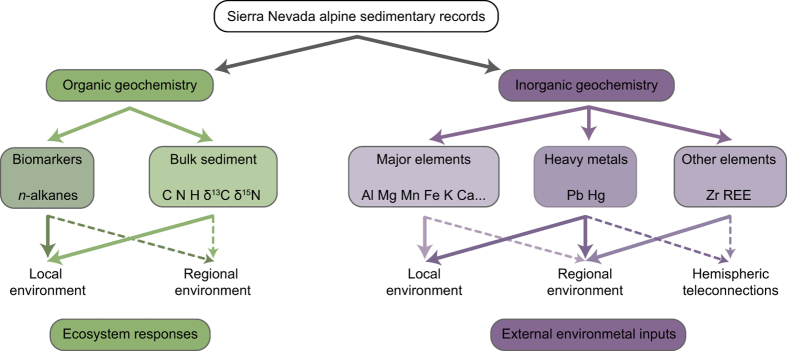
Schematic overview of the environmental proxies analysed in Sierra Nevada alpine wetlands. Solid lines represent environmental signals with high influence in the proxies (high sensitivity to these signals); dashed lines represent environmental signals with medium influence in the proxies (moderate sensitivity to these signals). Figure created by A. Garcia-Alix using Adobe Illustrator [5.5] (https://www.adobe.com/).

Previous paleoecological studies conducted in Sierra Nevada alpine areas have mostly focused on regional environmental and climate evolution within the context of the western Mediterranean climate domain^7,11–14^. In this respect, some inorganic geochemical records preserved at these elevations are extraordinary archives for tracking past regional and north-hemispheric scale teleconnections (e.g., Zr content, and La/Lu ratio)^4,6,15^. On the other hand, despite the Pb and Hg deposition occurs widely throughout the Northern Hemisphere, these metals also record local mining, metallurgy and industrial atmospheric pollution sources^4,16^. Local alpine environmental conditions in these sites can be specifically reconstructed by means of these and other inorganic elements related to catchment evolution, as well as organic bulk sediment and biomarker proxies that evidence past local biogeochemical cycles^4–7,17^ (Fig. 1).

The most important factors controlling the local biogeochemical behaviour in Sierra Nevada alpine wetlands are: 1) the length of the ice free season, which typically extends from May to October^4,17–19^, 2) the water availability, since Sierra Nevada is located in a semi-arid region^4,5^, and 3) the allochthonous nutrient inputs, as these wetlands are oligotrophic and their main nutrient input is via atmospheric deposition^18–20^. In addition, human activities have had isolated impacts on the sites, especially during the last hundred years^4,21^.

**Figure 2:**
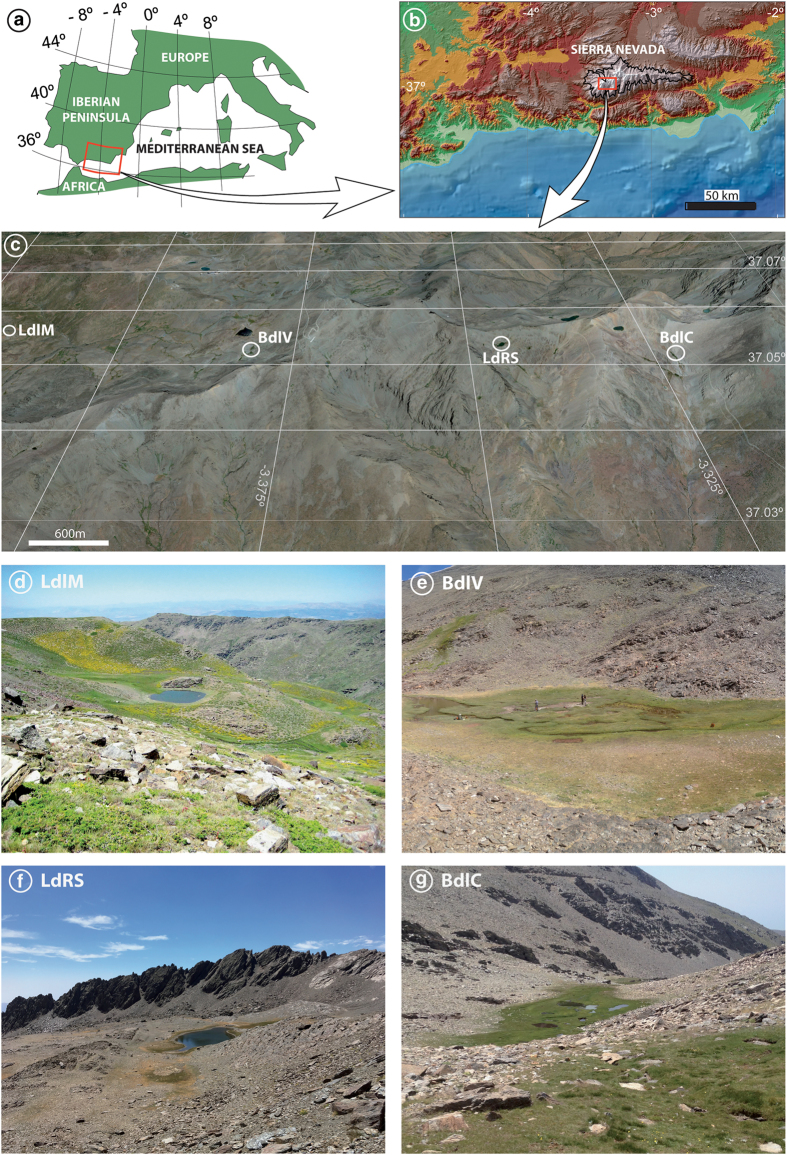
Geographical setting. (**a**) Studied area in the western Mediterranean region, and (**b**) location of the Sierra Nevada National Park (black line). (**c**) Situation of studied sites: Laguna de la Mula (LdlM), Borreguil de la Virgen (BdlV), Laguna de Río Seco (LdRS), and Borreguil de la Caldera site (BdlC). Detailed pictures of (**d**) Laguna de la Mula, (**e**) Borreguil de la Virgen, (**f**) Laguna de Río Seco, and (**g**) Borreguil de la Caldera. Data source and software: (**a-c**) modified from García-Alix et al. (2017)^4^, (**a**) map created by P. Ruano using Adobe Illustrator [5.5] (https://www.adobe.com/), (**b**) data from Suttle Radar Tomography Mission (SRTM-90: http://www2.jpl.nasa.gov/srtm/)^47^ plotted by means of ArcMap [10.1] (http://www.esri.com/software/arcgis/arcgis-for-desktop), (**c**) map from Google Earth Pro [7.1.5.1557] (https://www.google.es/earth/download/gep/agree.html) using the data provided by Google 2016 and DigitalGlobe 2016. **(d)** Picture from R.S. Anderson, **(e** and **g)** pictures from G. Jiménez-Moreno, **(f)** picture from A. García-Alix.

This data descriptor includes all the organic and inorganic geochemical data from previously studied Holocene sedimentary records that characterise past alpine wetland environments in Sierra Nevada. These data have been only partially published (~45% of the data) and come from four sites at different elevations, ranging from 2497 to 3020masl. They are, from west to east: Laguna de la Mula (LdlM), Borreguiles de la Virgen (BdlV), Laguna de Río Seco (LdRS), and Borreguil de la Caldera (BdlC) (Table 1; Fig. 2). Each of the sites, located in former glacial valleys or cirques, are within a 1.25 km^2^ area, with a maximum distance of ∼8km between the westernmost (LdlM) and the easternmost (BdlC) site (Fig. 2). Their catchment basins consist of bare mica-schist rocks without soil development and scarce vegetation (<20% in catchment surface)^10,15^ mainly concentrated around the water bodies. The main water bodies in the wetlands are shallow lakes without thermal stratification and an almost neutral pH (from 6 to 8)^17,22^. There are no available pH data from peaty areas. Vegetation mainly consists of graminoid-dominated (Cyperaceae and Poaceae) alpine meadows, although bryophytes predominate in the wetland-pond transitions. Vegetation distribution in Sierra Nevada is primarily controlled by precipitation and temperature, determining elevational belts. Only LdlM occurs near the local tree line (∼2500masl). The other records are in the tundra-like zone above ∼2900masl^11,23^.

## Methods

A multi-proxy approach based on geochemical analyses has been developed in four sedimentary cores collected in two peat bogs and two shallow lakes facing different hillslopes (Table 1). To track the source of the organic matter in the sediments several indices in bulk sediment samples have been selected: total organic carbon (TOC), total nitrogen (TN), total hydrogen (TH), atomic hydrogen – carbon ratio (H/C), atomic carbon – nitrogen ratio (C/N), and carbon and nitrogen isotopes^24^. The organic matter has been also characterised more specifically by means of leaf wax (*n*-alkanes) indices^24^. In this regard, the length of the carbon chain in *n*-alkanes can be related to different kinds of vegetation in the catchment basins, as well as potential water stress: short *n*-alkanes are related to aquatic environments, and long *n*-alkanes usually to terrestrial plants in the extreme environments of Sierra Nevada^4^. So, three *n*-alkane indices from leaf wax biomarkers, assessing the length of the carbon chain length, are used to constrain the source of organic matter and the water availability in the environments: the average chain length (ACL), the portion aquatic (Paq)^25^, and the carbon preference index CPI^26^. The potential detrital and aeolian input in these areas are depicted by means of La/Lu^6,27,28^ (sources of N African aeolian dust), Zr/Th, and Zr/Al ratios (amount of N African aeolian inputs)^4,6,29^ as well as Mg/Al (catchment basin runoff), among others^6,30^. Mn/Al ratios are usually related to the redox conditions in aquatic environments^31^; nevertheless, the complex Mn behaviour makes the reconstruction of oxygen conditions difficult based solely on this proxy. The anthropogenic heavy metal atmospheric pollution at these high elevation wetlands can be tracked by means of the Pb, Pb/Al and Hg records^4,6,32^. All these raw data along with other unpublished geochemical data are specified in the datasets (Data Citation 1).

### Sampling methods, sediment cores and age models

Four sedimentary records were extracted in the studied areas from 2006 to 2013 using a Livingstone piston corer and an Aquatic Research corer. They are named according to the year when they were retrieved and the number of cores extracted: Laguna de la Mula, LdlM 10-02; Borreguiles de la Virgen, BdlV 06-01; Laguna de Río Seco, LdRS 06-01 and LdRS 06-02; and Borreguil de la Caldera, BdlC 13-01. Their lengths were: 32.5 cm (LdlM 10-02), 169 cm (BdlV 06-01), 150 cm (LdRS 06-01+LdRS 06-02: LdRS 06-02-uppermost 10cm; LdRS 06-01- 140 cm), and 56 cm (BdlC 13-01). Only one drive was retrieved from cores LdlM 10-02, BdlC 13-01, and LdRS 06-02 and four and seven drives were retrieved in cores LdRS 06-01 and BdlV 06-01, respectively. Drives 01 and 02 from BdlV 06-01 and drive 01 from LdRS 06-01 were compacted during drilling and the real coring depth in these cases has been reconstructed. This is specified in each file (Data Citation 1). Sediment samples for the different analyses were taken from the cores at different resolution, depending on the proxy studied (Table 2). Age models from the cores were computed using ^14^C ages in LdlM 10-02^7^, BdlV 06-01^12^, LdRS 06-01^11^, and BdlC 13-01^33^ as well as Cs-Pb (LdRS 06-02)^11^ by means of Clam package (http://www.chrono.qub.ac.uk/blaauw/clam.html)^34^ for R open-source software (https://www.r-project.org/) and the calibration curves IntCal09^35^ and IntCal13^36^ (Table 3).

### Organic geochemistry

#### Elemental analyses in bulk sediment

Pre-weighted and freeze-dried samples were decarbonated overnight by means of acid digestion (HCl 1M). The acid concentration was 1M because carbonate content in the samples was low. When all the carbonate was digested, the solution was centrifuged to remove the acid, and samples were rinsed with Milli-Q water and centrifuged five times. After reaching a neutral pH, the obtained carbonate-free product was freeze-dried again. When samples were totally dry, they were split in two aliquots: one for elemental analyses and another one for C and N isotope analyses. The elemental composition of the samples was measured using a Thermo Scientific Flash 2000 elemental analyser with He as carrier gas at the Centre for Scientific Instrumentation of the University of Granada, Spain (hereafter CIC-UGR). A flash combustion was produced at 1000 ºC, and the obtained gas, after passing through a reduction column with Cu, was separated by means of a chromatographic column and quantified with a Thermal Conductivity Detector CTD (Data Citation 2, Data Citation 3, Data Citation 4 and Data Citation 5).

#### Carbon and Nitrogen analyses in bulk sediment

C and N isotopes were measured in the other aliquot of the decarbonated bulk samples by means of isotope-ratio mass spectrometry (IRMS) with a coupled elemental analyser (EA). In this case, the obtained gases from the EA (N_2_ and CO_2_) were analysed in the IRMS in order to obtain their isotopic composition. We used two different configurations: Carlo Erba Ba 1500 series 2 Elemental Analyser attached to a Thermo Finnigan Delta plus XL IRMS (Instituto Andaluz de Ciencias de la Tierra CSIC-UGR, Spain) in samples from LdlM, BdlV, and LdRS, and an Euro EA 300 Elemental Analyser attached to an Isoprime 50 V IRMS (CIC-UGR) in samples from BdlC. The isotopic measurements were calibrated using internal and international standards (see Technical Validation section), and expressed using the δ notation, which relates the isotopic abundance of an element in the sample and that of the same element in a reference material: δ ‰= [(R_sample_/R_Ref_)−1]x1000. This reference is VPDB in the case of δ^13^C and AIR, in the case of δ^15^N (Data Citation 2, Data Citation 3, Data Citation 4 and Data Citation 5).

#### Specific compound analyses

Pre-weighted, homogenized and freeze-dried samples were dissolved by means of sonication (20 min) and temperature (38 °C for 1 hour) using DCM:MeOH (3:1) solution. The supernatant solvent was collected after centrifuging at 3300 rpm and dried in a nitrogen stream. These steps were repeated at least two more times to make sure that all the lipids had been extracted from the samples. The neutral fraction of this total lipid extract was obtained by means of aminopropyl-silica gel chromatography and a solution of 1:1 DCM:isopropanol. Afterwards, the aliphatic hydrocarbon fraction, with the *n-*alkanes, was extracted using the elution of the neutral fraction with hexane trough a 230–400 mesh/35–70micron silica-gel chromatographic column. Finally, the *n-*alkanes were analysed at the BECS laboratory (University of Glasgow, UK) by means of a GC-FID (Shimadzu 2010) in order to quantify them, and a GC-MS (Shimadzu OP2010-Plus Mass Spectrometer interfaced with a Shimadzu 2010 GC) in order to identify the compounds of the most complicated samples (Data Citation 3, Data Citation 4 and Data Citation 5).

### Inorganic geochemistry

#### Inductively Coupled Plasma Mass Spectrometry and Atomic Absorption Analyses

About 0.1–0.2 g of sediment samples were dissolved using HNO_3_ (65% Panreac PA-AR)+HF (40% Suprapur) in Teflon-lined vessels at high temperature and pressure during 150 min. Afterwards, they were completely evaporated and re-dissolved in 100 ml of 4 vol.% HNO_3_. This solution was split in two aliquots. One was analysed by means of inductively coupled plasma mass spectrometry (ICP-MS) using a Perkin Elmer Sciex Elan 5000 (for Li, Rb, Cs, Be, Sr, Ba, Sc, V, Cr, Co, Ni, Cu, Zn, Ga, Y, Nb, Ta, Hf, Mo, Sn, Tl, U, Ce, Pr, Nd. Sm, Eu, Gd, Tb, Dy, Ho, Er, Tm, Yb, Lu, Zr, Pb, Th, and La). The other aliquot was analysed by means of flame Atomic Absorption (AAS) using a Perkin-Elmer 5100 ZL spectrometer with an analytic error of 2% (for Al, Mn, Ca, Fe, Mg, and K). Two different flames were used: one of acetylene/nitrous oxide for the determination of Al and another of acetylene/air for the other elements. These inorganic analyses were conducted at the CIC-UGR. Although row data are expressed in ppm, the concentration of some selected paleoenvironmental proxies (Zr, Mg, Mn, Pb, among others) are normalised by refractory elements (i.e., Al, or Th, in this case)^37,38^ in order to correct the dilution caused by sedimentary barren phases of a particular element^39,40^ (Data Citation 6).

#### X-Ray fluorescence Scanner analyses

High-resolution elemental profiles (Al, Si, S, K, Ca, Ti, Fe, Zr, Br, Rb, and Sr) at the BdlC core were obtained by means of an Avaatech X-Ray fluorescence (XRF) core Scanner at the XRF-Core Scanner Laboratory (University of Barcelona, Spain). The core was scanned two times with a point sensor: one at 10 s count time (10 kV X-ray voltage and 650 mA X-ray current for light elements, such as Al, Si, S, K, Ca, Ti, and Fe), and another one at 35 s count time (30kV X-ray voltage and 1700 mA X-ray current for heavy elements, such as Zr, Br, Rb, Sr). Triplicate measurements were analysed every 25 analyses. Results were expressed in intensities (counts per second, cps) as well as normalized for the total sum of cps in every measure in order to avoid the influence of the water content and the sediment surface conditions (Data Citation 7).

#### Mercury analyses

Total mercury concentrations were determined using an Advanced Mercury Analyser (LECO AMA-254) with an absolute mass detection limit of 0.01 ng of Hg, following analogous procedures to those reported by Diez et al., 2007^41^ at the Institute of Environmental Assessment and Water Research (IDAEA-CSIC, Spain). This instrumentation, originally developed by Altec, Ltd., Czech Republic, is a single-purpose atomic absorption spectrometer for determination of mercury traces in solid and liquid specimens without sample pre-treatment or pre-concentration. Sediment samples and quality control materials with masses of 20 mg to 100 mg were automated, introduced into a quartz combustion tube in a nickel boat and dried at 120 °C for 50 s. Subsequently, the instrument self-seals the tubes. Afterwards, samples were combusted in an oxygen-rich atmosphere (99.5%) and the released gasses were transported using oxygen as carrier gas through specific catalytic converter (a Mn_3_O_4_/CaO-based catalyst at 750 ºC), in order to obtain a complete oxidation as well as the retention of halogens, nitrogen, and sulphur oxides. As a consequence, the different mercury species are converted into elemental Hg vapour, which is collected in a gold-plated ceramic amalgamator. Subsequently, the mercury is released by means of an oxygen flush for 150 s and the amalgamator heating up to approx. 700 °C. The gas is driven to a cuvette at ~120 °C in order to prevent condensation and to minimize potential carry-over effects. The source was a low-pressure mercury vapour lamp at of 253.65 nm wave-length, and a detector, with a working range between 0.05 ng and 500 ng, acquired the signal. Data are expressed in ppb (Data Citation 8).

### Code availability

The database includes seven datasets stored in seven files (Data Citation 1). The files with the different datasets are named with the acronym of the site and the data source; i.e. BdlV_organic. The files and the information they contain are listed in Table 2. Each data file includes the following fields for each sample:

*Core ID*

*Drive*

*Top sampling depth*

*Bottom sampling depth*

*Top real depth*

*Bottom real depth*

*Mean real depth*

*Top age Cal yr BP*

*Bottom age Cal yr BP*

*Mean age Cal yr BP*

*Proxy#1*

*Proxy#2*

*Proxy#n-n+1*

Data from XRF scanner in the file *BdlC_inorganic* do not contain the fields: *bottom sampling depth, bottom real depth, mean real depth, bottom age Cal yr BP* and *mean age Cal yr BP* since measurement were taken with a point sensor in specific locations (*top sampling depth*).

Proxy units are showed between brackets when are needed. Each single data can be named as follows: core-ID#top_real_depth/top_age#proxy, i.e., *BdlV 06-01#10 cm/155 cal yr BP#δ*^*13*^*C.* New paleogeochemical data can be easily added to this database when they are available so that this database will always be updated with the latest geochemical findings in these sites.

## Data Records

The dataset presented in this paper shows information of the organic and inorganic content from four alpine sites at the Sierra Nevada National Park: LdlM, BdlV, LdRS, and BdlC (Table 1). These geochemical records have different lengths, registering the environmental evolution of these shallow lakes and peatlands from the last 4.1 ky (LdlM, the shortest sedimentary record in the area) to the last 12–12.5 ky (LdRS, the longest sedimentary record in the area) (Table 1).

Regarding the organic data, we present the total organic carbon (TOC), total nitrogen (TN), total hydrogen (TH), atomic hydrogen–carbon ratio (H/C), atomic carbon–nitrogen ratio (C/N), carbon isotopic composition (δ^13^C), nitrogen isotopic composition (δ^15^N), and several *n-*alkane indices (average change length, ACL; portion aquatic, P_aq_; carbon preference index, CPI), as well as the *n*-alkane concentration in each sample. ACL, CPI and P_aq_ have been worked out from the *n-*alkane concentrations (C_xx_) following these equations:

*ACL*^42^=(25xC_25_+27xC_27_+29xC_29_+31xC_31_ +33xC_33_)/ (C_25_+C_27_+C_29_+C_31_+C_33_) after Poynter and Eglinton (1990).

*CPI*^43,44^=0.5 x[(C_25_+C_27_+C_29_+C_31_+C_33_)/(C_24_+C_26_+C_28_+C_30_+C_32_)+(C_25_+C_27_+C_29_+C_31_+C_33_)/(C_26_+C_28_+ C_30_+C_32_+C_34_)] after Bray and Evans (1961).

*P*_*aq*_^25^= (C_23_+C_25_)/ (C_23_+C_25_+C_29_+C_31_) according to Ficken et al. (2000).

The inorganic geochemical data available are the concentrations of Al, Si, Mn, Ca, Fe, Mg, K, S, Li, Rb, Br, Cs, Ti, Be, Sr, Ba, Sc, V, Cr, Co, Ni, Cu, Zn, Ga, Y, Nb, Ta, Hf, Mo, Sn, Tl, U, Ce, Pr, Nd. Sm, Eu, Gd, Tb, Dy, Ho, Er, Tm, Yb, Lu, Zr, Pb, Th, and Hg, as well as the ratios Mg/Al, Mn/Al Zr/Th, Zr/Al, La/Lu, and Pb/Al (Table 2).

All of these data are related to 1) the external mechanisms that generated the sedimentary record: i.e. runoff, aeolian input/atmospheric deposition, or the redox conditions in water environments, which are mainly related to the inorganic geochemical data, that eventually were boosted either by climate/environmental or indirect human influence; and 2) the environmental responses of these extreme environments to the climate/environmental and human pressures (mostly organic geochemical data) (Fig. 1). We present all these raw data in the datasets (Data Citation 1); however, the interpretation of these data in the context of our research can be found in the original publications^4–7,21^.

## Technical Validation

### Organic geochemistry

#### Elemental analyses in bulk sediment

The equipment was calibrated every day using a certified Sulfanilamide standard, whose elemental composition is: N 16.27%, C 41.84%, H 4.68%, and S 18.62%. The calculated precision of the measurements was better than ±0.1%. The CIC-UGR works under a Quality Management System following the requirements of the UNE-EN-ISO-9001, which certifies the technical quality of the obtained data.

#### Carbon and Nitrogen analyses in bulk sediment

The analyses that were conducted in the EA-Thermo Finnigan DELTA plus XL IRMS used four internal standards EEZ14 -phthalic acid- (δ^13^C: -30.63‰ VPDB) and EEZ 21 -sucrose- (δ^13^C: -11.65‰ VPDB), EEZ17 -urea Merk- (δ^15^N: −1.02‰ air), and EEZ23 -shark cartilage- (δ^15^N: 16.01‰ air), contrasted with the IAEA international references NBS-22-oil- (δ^13^C: −30.03‰ VPDB), IAEA-CH-6 -sucrose- (δ^13^C: −10.45‰ VPDB), and IAEA-N1 -ammonium sulphate- (δ^15^N: +0.4‰ air). The calculated precision of the measurements was better than ±0.1‰ for δ^13^C and δ^15^N.

The analyses that were conducted in the EA-Isoprime 50 V IRMS used Certified Elemental Microanalysis standards: Sorgo Flour Standard (δ^13^C: −13.68‰ VPDB and δ^15^N: +1.58‰ air), Wheat Flour Standard (δ^13^C: −27.1‰ VPDB and δ^15^N: +2.85‰ air), and Casein Standard (δ^13^C: −26.98‰ VPDB and δ^15^N: +5.94‰ air). These standards were calibrated to the international standards IAEA-CH-6 sucrose- (δ^13^C: −10.45‰ VPDB) and IAEA-N1 -ammonium sulphate- (δ^15^N: +0.4‰ air). The calculated precision of the measurements was better than ±0.1‰ for δ^13^C and δ^15^N. The analyses were conducted under a Quality Management System following the requirements of the UNE-EN-ISO-9001 at the CIC-UGR.

#### Specific compound analyses

The reproducibility of the measurements was checked by means of an external standard with a mixture of *n-*alkanes (C_16_, C_18_, C_19_, C_20_, C_23_, C_25_, C_26_; C_28_; C_30_, C_32_, C_37_) measured every five samples. The standard reproducibility was better than 97%. The concentration of the *n-*alkanes was worked out with the C25 *n-*alkane of the same external alkane mixture mentioned above. The concentration of this C25 *n-*alkane was 10 μg/ml.

### Inorganic geochemistry

#### ICP analyses

Each sample was measured in triplicate. Re and Rh (25 ppb) internal standards (25 ppb) were used to test the performance of the equipment. In addition, data were contrasted with several reference geo-standards: UBN, PMS, WSE, BEN, BR, AGV, DRN, GSN GA and GH^45^. The instrumental errors during the measurement of the sample batches were±2% for elemental concentrations >50 ppm and±5% for concentrations between 50 to 5 ppm^46^. The technical validation of the analyses is certified by the Quality Management System of the CIC-UGR that follows the requirements of the UNE-EN-ISO-9001.

#### Atomic Absorption analyses

The Perkin-Elmer 5100 spectrometer has an analytic error lower than 2%. Certified Perking Elmer standards for AA (ISO Guide 34 and ISO 17025 – certified by A2LA) at a concentration of 1000 μg/m in a solution of 2% of HNO_3_ were used for each element. Blank samples were measured for each element to establish their detection limit, which was: <190 ppm (Mn), <5 ppm (Al), <200(Ca), <5 ppm (Fe), <340 ppm (Mg), and <190 ppm (K). The analyses were conducted following the UNE-EN-ISO-9001 requirements of the Quality Management System at the CIC-UGR.

#### X-Ray fluorescence Scanner analyses

Measurements of the SARM4 standard of the National Institute of Standards and Technology (NIST) were performed in order to test the stability of the X-ray tube at the beginning and at the end of the measurement session every day. In addition, samples were measured in triplicate every 25 analyses. The % mean error of the measurements [(stdes/mean)*100] was 3.5% (Al), 0.9% (Si), 1.4% (S), 1.1% (K), 2.6% (Ca), 1.4% (Ti), 0.6% (Fe), 2.5% (Zr), 4.3% (Br), 4.4% (Rb), 2.8% (Sr).

#### Mercury analyses

The absolute mass detection limit of the LECO AMA-254 was 0.01 ng of Hg. The entire analytical procedure was validated by analysing certified reference material DORM-3 (Fish tissue, NRCC, Canada) at the beginning and end of each set of samples, ensuring that the instrument remained calibrated during the course of the analytical routine.

## Additional information

**How to cite this article:** García-Alix, A. et al. Holocene geochemical footprint from Semiarid alpine wetlands in southern Spain. *Sci. Data* 5:180024 doi: 10.1038/sdata.2018.24 (2018).

**Publisher’s note:** Springer Nature remains neutral with regard to jurisdictional claims in published maps and institutional affiliations.

## Supplementary Material



## Figures and Tables

**Table 1:** Main features of the studied sites.

**Site**	**Type**	**Record length (cm -- ky)**	**Location**	**Elevation (masl)**	**Surface (ha)**	**Orientation**	
					**Site**		**Catchment**
LdlM	Lake	32.5 cm 4.1 ky	37°3’35’’N 3°25’01’’W	2497	0.10	25	NW
BdlV	Bog (present) Lake (early –middle Holocene)	169 cm 8.5 ky	37º03’10’’N 3º22’43’’W	2945	0.18	30	NW
LdRS	Lake	150 cm 12.0 -12.5 ky	37º03’08’’N 3º20’44’W	3020	0.42	9.9	S
BdlC	Bog	56 cm 4.5 ky	37º03’02’’N 3º19’24’’W	2992	0.17	62	S
Acronyms: Laguna de la Mula (LdlM), Borreguil de la Virgen (BdlV), Laguna de Río Seco (LdRS), Borreguil de la Caldera (BdlC).							

**Table 2:** Organic and inorganic analyses conducted in the sedimentary cores as well as number of samples measured with the different techniques and the file name where these data are stored (Data Citation 1).

**Site**	**Organic analyses**				**Inorganic analyses**								
	**Elemental Analyser**				**EA-IRMS**		**GC-FID**	**Data files in Data Citation**	**AAS**	**ICP-MS**	**Mercury Analyser**	**XRF scanner**	**Data files in Data Citation**
	**TOC, TN**	**TH**	**C/N**	**H/C**	**δ**^**13**^**C**	**δ**^**15**^**N**	***n-*****alkanes**				Hg		
LdlM	31		31		31	31		LdlM_organic (Data Citation 2)					
BdlV	73		79		79	74	93	BdlV_organic (Data Citation 3)					
LdRS	68	68	68		68	68		LdRS_organic (Data Citation 4)	68	68			LdRS_inorganic (Data Citation 6)
BdlC	81	81	82	82	56	51	50	BdlC_organic (Data Citation 5)			18	78	BdlC_inorganic (Data Citation 7) BdlC_Hg: (Data Citation 8)
AAS data: Al, Mn, Ca, Fe, Mg, and K; ICP-MS data: Li, Rb, Cs, Be, Sr, Ba, Sc, V, Cr, Co, Ni, Cu, Zn, Ga, Y, Nb, Ta, Hf, Mo, Sn, Tl, U, Ce, Pr, Nd. Sm, Eu, Gd, Tb, Dy, Ho, Er, Tm, Yb, Lu, Zr, Pb, Th, and La; XRF-scanner data: Al, Si, S, K, Ca, Ti, Fe, Zr, Br, Rb, and Sr. Acronyms: EA, Elemental Analyser; IRMS, Isotope-ratio mass spectrometry; GC-FID, Gas Chromatography with Flame-Ionization Detection; AAS, Atomic Absorption; ICP-MS, Inductively coupled plasma mass spectrometry; XRF-scanner, X-ray fluorescence scanner.													

**Table 3:** Age data from the studied cores

**Laboratory Code**	**Core**	**Depth (cm)**	**Material Dated**	**Dating Method**	^**14**^**C age (yr BP)**	**SD (±)**	**Calibrated Age (cal yr BP/AD)**
Reference age	LdlM 10-02	0.0		Present			−60
DirectAMS-1203-006	LdlM 10-02	2.5	OBS	^14^C	834	19	739
DirectAMS-1203-007	LdlM 10-02	9.5	OBS	^14^C	2038	24	1990
DirectAMS-1203-008	LdlM 10-02	14.5	OBS	^14^C	2535	28	2624
DirectAMS-1203-009	LdlM 10-02	18.0	OBS	^14^C	2887	20	3018
DirectAMS-1203-010	LdlM 10-02	22.0	OBS	^14^C	3397	20	3650
*DirectAMS-1203-011*	*LdlM 10-02*	*27.5*	*OBS*	^*14*^*C*	*3913*	*22*	*4356*
UCIAMS81595	LdlM 10-02	30.5	OBS	^14^C	3720	20	4042
Reference age	BdlV 06-01	0.0		Present			−56
UCIAMS-51248	BdlV 06-01	34.5	VR	^14^C	730	15	675
UCIAMS-69120	BdlV 06-01	44.2	VR	^14^C	3220	20	3428
*UCIAMS-67124*	*BdlV 06-01*	*47.5*	*VR*	^*14*^*C*	*5435*	*25*	*6240*
*UCIAMS-67125*	*BdlV 06-01*	*53.96*	*VR*	^*14*^*C*	*5000*	*20*	*5722*
UCIAMS-67126	BdlV 06-01	61.8	VR	^14^C	3960	20	4430
UCIAMS-51249	BdlV 06-01	72.4	VR	^14^C	4395	15	4941
UCIAMS-51250	BdlV 06-01	100.0	VR	^14^C	5410	15	6241
Beta-22171	BdlV 06-01	144.0	VR	^14^C	6470	40	7375
UCIAMS-51251	BdlV 06-01	159.0	VR	^14^C	7245	20	8052
Reference age	LdRS 06-02	0.0		Present			−56
USC-LdRS 06-02-1	LdRS 06-02	5.0	BS	^137^Cs			1963 AD
USC-LdRS 06-02-2	LdRS 06-02	15.0	BS	^210^Pb			1891 AD
UCIAMS-51255	LdRS 06-01	20.0	VR	^14^C	1520	15	1398
UCIAMS-63003	LdRS 06-01	26.75	VR	^14^C	2255	20	2234
UCIAMS-51256	LdRS 06-01	40.0	VR	^14^C	3060	15	3295
UCIAMS-63004	LdRS 06-01	46.0	VR	^14^C	3525	20	3786
UCIAMS-51257	LdRS 06-01	60.0	VR	^14^C	4010	15	4480
UCIAMS-51258	LdRS 06-01	80.0	VR	^14^C	5450	30	6246
UCIAMS-63005	LdRS 06-01	83.25	VR	^14^C	5505	20	6298
UCIAMS-63006	LdRS 06-01	109.5	VR	^14^C	6550	20	7453
UCIAMS-32495	LdRS 06-01	123.5	VR	^14^C	8570	60	9540
Reference age	BdlC 13-01	0.0		Present			−63
DirectAMS-004385	BdlC 13-01	13.7	VR	^14^C	388	24	469
DirectAMS-004386	BdlC 13-01	23.2	VR	^14^C	474	26	517
DirectAMS-004387	BdlC 13-01	36.8	VR	^14^C	1036	31	950
DirectAMS-004388	BdlC 13-01	46.4	VR	^14^C	2563	30	2725
DirectAMS-004389	BdlC 13-01	56.0	VR	^14^C	4066	29	4551
^14^C ages were calibrated using IntCal09 curve^35^ in LdlM^7^, LdRS^11^, and BdlV^12^, and IntCal13 curve^36^ in BdlC^33^. Dates in italics: old carbon ages not used in the age model. Acronyms: OBS, organic bulk sediment; BS, bulk sediment; VR, vegetal remains; DirectAMS#, Accium BioSciencies, Seattle, USA; Beta#, Beta Analytic, Inc. Miami, USA; UCIAMS#, University of California at Irvine W.M. Keck Carbon Cycle Accelerator Mass Spectrometry Laboratory, Irvine, USA. USC, University of Southern California, Los Angeles, USA.							
